# Desmoid Tumors: A Clear Perspective or a Persisting Enigma? A Case Report and Review of Literature

**DOI:** 10.14740/wjon961w

**Published:** 2016-04-03

**Authors:** Debi Prasad Mishra, Suman Saurav Rout

**Affiliations:** a Department of Pathology, MKCG Medical College and Hospital, Berhampur, Odisha, India; b Department of General Surgery, Institute of Medical Sciences and SUM Hospital, Bhubaneswar, Odisha, India

**Keywords:** Desmoid, Mesenchymal tumors, Familial adenomatous polyposis, Gardner’s syndrome, Adenomatous polyposis coli mutation

## Abstract

Desmoid tumors are benign but locally aggressive tumors of mesenchymal origin which are poorly circumscribed, infiltrate the surrounding tissue, lack a true capsule and are composed of abundant collagen. History of trauma or surgery to the site of tumor origin is elicited in up to one in four cases and they most commonly develop in the anterior abdominal wall and shoulder girdle but they can arise in any skeletal muscle. The clinical behavior and natural history of desmoid tumors are unpredictable and management is difficult with many issues remaining controversial, mainly regarding early detection, the role, type and timing of surgery and the value of non-operative therapies. We report a case of anterior abdominal wall desmoid tumor in a 40-year-old male with a previous history of surgery.

## Introduction

Desmoid tumors are cytologically bland fibrous neoplasms originating from the musculoaponeurotic structures throughout the body. The term desmoid, coined by Muller in 1838, is derived from the Greek word *desmos*, which means tendon-like. Desmoid tumors are monoclonal fibroblastic proliferations arising in musculoaponeurotic structures. They are benign but aggressive tumors of mesenchymal origin, forming a heterogenous group of pathologic entities resulting from the proliferation of well-differentiated fibroblasts [1, 2]. At microscopy, desmoid tumors are poorly circumscribed, infiltrate the surrounding tissue, lack a true capsule and are composed of abundant collagen surrounding poorly circumscribed bundles of elongated, slender, spindle-shaped cells of uniform appearance [3].

Most desmoid tumors occur sporadically but about 2-5% commonly occur in the abdominal cavity or abdominal wall in association with familial adenomatous polyposis (FAP) [4]. Inheritance (or new mutation) of one copy of adenomatous polyposis coli (APC) tumor suppressor gene is the cause of FAP and the two commonest causes of deaths in these patients are duodenal cancer and desmoid tumors [4]. In FAP-associated cases, desmoid tumors represent an extra-colonic manifestation of polyposis syndrome [5]. Every patient with desmoid tumor should therefore be evaluated for the presence of associated polyposis syndrome by taking a detailed family history, performing colonoscopy and possibly upper gastrointestinal (GI) endoscopy [6].

## Case Report

A 40-year-old male patient presented with a painless mass in the anterior abdominal wall for last 2 months. The size of the mass was gradually increasing in size to attain its present size. On examination, a firm non-tender mass of 5 × 6 cm was palpable. The mass was fixed to the anterior abdominal wall. He had no relevant positive family history. He had a history of a midline laparotomy performed for an abdominal trauma he had sustained during a road traffic accident 4 years earlier to the start of his symptoms. His blood parameters were within normal limits. Ultrasound of the abdomen revealed a large heterogenous mass with sharp delineated margins in the anterior abdominal wall. CECT of the abdomen revealed a well-circumscribed mass of size around 8 × 10 × 6 cm with attenuation similar to that of the abdominal musculature. Origin of the mass was traced to the rectus abdominis muscle. There was no involvement of any abdominal viscera, neither was there any evidence of adenopathy. Pre-operative FNAC also revealed features consistent with desmoid.

With an abdominal wall desmoid tumor in our minds, a wide surgical resection was performed with a peripheral healthy margin of about 3 cm. The defect in the anterior abdominal wall due to resection of the rectus muscle was repaired with polypropylene mesh. On cut section, the mass looked gritty and glistening with trabeculations similar to that seen in the scar tissue. On histopathological study, the tissue consisted of elongated spindle cells resembling fibroblasts embedded in collagenous matrix. The tumor cells seemed to invade the skeletal matrix distorting its architecture. The above histopathology strongly suggested towards a diagnosis of desmoid tumor which was again supported by immunohistochemistry study. The patient had an uneventful postoperative period.

## Discussion

### Pathophysiology and molecular insights

Although desmoid tumors most commonly arise from the rectus abdominis muscle in postpartum women and in scars due to abdominal surgery, they may arise in any skeletal muscle. Desmoid tumors tend to infiltrate adjacent muscle bundles, frequently entrapping them and causing their degeneration [7]. They may be derived from mesenchymal stem cells [8]. Although fixation to musculoaponeurotic structures is apparent, the overlying skin is normal. The myofibroblast is the cell considered to be responsible for the development of desmoid tumors.

Gardner’s syndrome or FAP is characterized by colorectal adenomatous polyps and soft and hard tissue neoplasms. The former may number in the hundreds to thousands. Gardner’s syndrome was regarded as a separate disease until the identification of the APC gene, at which point mutations in the APC gene were recognized as the underlying cause of both Gardner’s syndrome and FAP. Some authors regard Gardner’s syndrome as a subset of FAP, and some have even suggested that the term Gardner’s syndrome be replaced by FAP. Additionally, evidence also exists for a genetic predisposition to desmoid tumors in FAP, independent of the APC mutation.

Desmoid tumors occur at a rate of 10-15% in patients with FAP, an autosomal inherited disease caused by germline mutations in the APC gene. Sporadic forms have no hereditary background [9]. Desmoid tumors show biallelic APC mutation, with one change usually occurring distal to the second beta-catenin binding/degradation repeat of the gene (3' to codon 1399) [10, 11]. The relationship between extracolonic manifestations and the site of the APC mutation suggests a specific role of the APC protein in different tissues. However, unknown genetic factors independent of APC may be important in the susceptibility to desmoid tumors in patients with FAP.

In desmoid tumors, one of the two mutations usually occurs distal to the second beta-catenin binding/degradation repeat of the gene (3' to codon 1399). Catenin and catenin-binding genes have been found to be associated with neoplastic processes in a number of ways. Independent predictors of increased desmoid risk in one study were said to be 1) germline mutation distal to codon 1399, 2) any family history of gastrointestinal disease, and 3) a strong family history of desmoid tumors.

The relationship between certain extracolonic manifestations and sites of the APC mutation suggests specific roles of the APC protein in different tissues. These different roles may correspond to specific sites of missense mutations in the APC gene. For example, dental manifestations of Gardner’s syndrome have been suggested to be associated with mutations at or near codon 1556. However, the influence of unknown genetic factors independent of APC in susceptibility to desmoid tumors in FAP needs to be explored.

FAP results from a germline mutation in the APC gene. Desmoid tumors are associated with a biallelic APC mutation in the affected tissue. This usually results from a spontaneous somatic mutation in the unaffected APC gene of a single cell in a patient with the FAP syndrome. This process is an example of the Knudsen “two hit” hypothesis, in which a tumor suppressor gene, such as APC, must be biallelically mutated in order for a specific type of tumor to occur.

In genetically normal individuals, with normal germline genes, this necessitates a rare combination of events, such that at least two somatic mutations must occur in both alleles of a single tumor suppressor gene, in this case the APC gene. In FAP syndrome patients, one APC germline gene is already mutated in every cell in the body (barring a rare reverse somatic mutation in some cells), and, therefore, only one new somatic mutation is required in the opposite APC gene for the tumor to develop.

FAP may be associated with mutations in the APC gene, but mutations in several other genes, particularly mismatch DNA repair genes, which are primarily responsible for ensuring integrity of polymerases responsible for DNA replication, may also result in familial colonic polyposis. These patients with familial colonic polyposis typically do not show other manifestations of Gardner’s syndrome. Conversely, extracolonic manifestations characteristic of Gardner’s syndrome may occur independent of intestinal polyps or a mutation in the APC gene. Nuclear localization of β-catenin may be evident in pediatric desmoids regardless of mutation status, with most showing somatic mutations in CTNNB1 [12]. However, many harbor germline mutations in APC. CTNNB1 mutations are common in sporadic desmoid tumors [13].

### Epidemiology

Overall, desmoid tumors are reported to account for 0.03% of all neoplasms [14]. When present in patients with familial polyposis of the colon, the prevalence of desmoid tumors is as high as 13% [15]. Despite their benign histologic appearance and negligible metastatic potential, the tendency of desmoid tumors to cause local infiltration is significant in terms of 1) deformity, morbidity, and mortality resulting from pressure effects and 2) potential obstruction of vital structures and organs.

Desmoid tumors most commonly occur in women after childbirth. Desmoid tumors are twice as common in females as in males; however, 60 patients were described [16], and the female-to-male ratio was 1.2:1. In children, the sex incidence is equal.

Although desmoid tumors are more common in persons aged 10 - 40 years than in others, they do occur in young children and older adults. Sixty patients were described by Lee et al in 2006, with an average age at diagnosis of 41.3 years [16].

Trauma, such as a prior surgical incision, has also been found to be associated with occurrence of these tumors and is particularly pronounced in the setting of FAP. This association is of interest as desmoid tumors share many morphologic features in common with scar tissue, particularly hypertrophic scar.

### Clinical presentation

Desmoid tumors most commonly arise in the anterior abdominal wall and shoulder girdle, although they can occur in any skeletal muscle. A retroperitoneal presentation is common with FAP and Gardner’s syndrome. A history of trauma to the site (mostly surgical) is elicited in one in four cases [17]. There have also been reported cases of implant-associated breast desmoid tumors [18].

Desmoid tumors usually present as a slowly growing solitary firm mass with ill-defined margins and no distinct capsule, but multifocal lesions confined to the same anatomical region can occasionally be encountered. While they can arise anywhere in the body, most desmoids develop in the extremities and superficial trunk wall. When arising from intra-abdominal structures, desmoids can obstruct small bowel or ureters or compress neural or vascular structures causing digestive, motor or perfusion deficits.

They may be intra-abdominal (in the abdominal wall, especially the rectus and internal oblique muscles with their fascial coverings, and mesentery or retroperitoneum), extra-abdominal (the shoulder girdle, trunk, and lower extremities), multiple familial, and as part of Gardner’s syndrome.

### Evaluation

Correlation with the clinical presentation of the tumor is usually extremely helpful in narrowing the differential diagnosis, specifically the size and depth of the lesion. A histological confirmation obtained via a core needle or incisional biopsy is necessary for accurate diagnosis.

The preferred diagnostic test is excisional biopsy of the tumor. A fine-needle aspiration biopsy specimen may be considered before going for a surgical excision. Fine-needle aspiration is fairly reliable for recognition of the benign nature of desmoids. Occasional over- and under-diagnosis of malignancy can occur; however, core needle biopsy appears to be more reliable. Electron microscopy may be performed.

**Figure 1:**
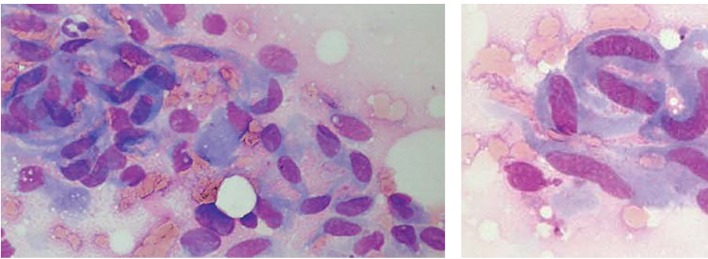
Fine-needle aspirates of desmoid tumors showing coherent clusters of uniform spindle cells with abundant cytoplasm and oval to elongated nuclei with evenly distributed chromatin. Large, basophilic multinucleate cells representing atrophic muscle fibers are also seen.

The fine-needle aspiration smears of desmoids had a fairly uniform appearance, differing from case to case mainly by a highly variable yield, with some smears having very scant material and others being strikingly cellular. The tumor cells had the characteristic features of fibroblastic/myofibroblastic cells, being spindle-shaped or polygonal and in most cases with fairly abundant basophilic cytoplasm. Poorly preserved spindle cells with stripped, “naked” oval nuclei were also seen in several cases. The oval nuclei had finely dispersed chromatin with no or few small nucleoli. Tumor cells occurred as single cells but they were often in coherent clusters, forming a vague fascicular pattern (Fig. 1). In addition to clusters of tumor cells, there were also fragments of a collagenized, finely fibrillar background matrix. In many cases, a striking feature was the occurrence of large, multinucleate cells representing atrophic muscle fibers.

**Figure 2:**
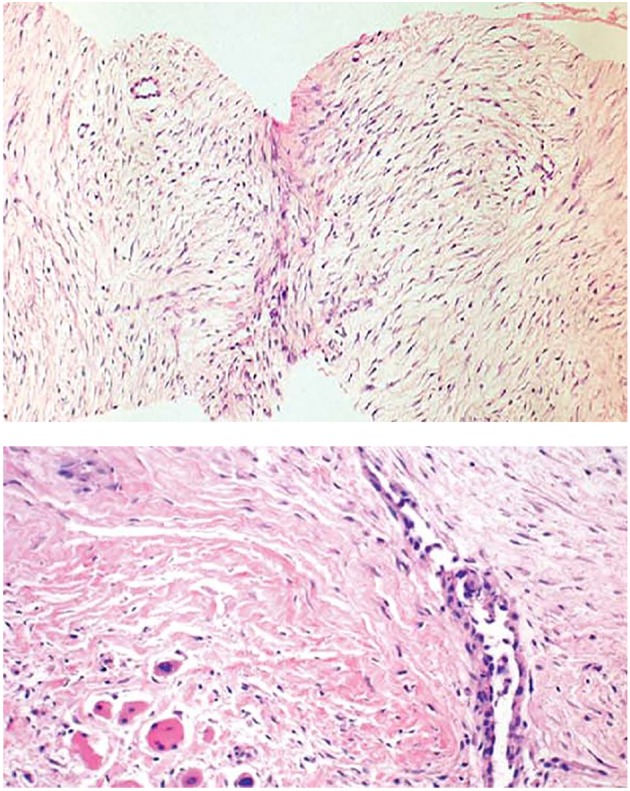
Core needle biopsies of desmoid tumors showing the characteristic histological features, including evenly distributed fibroblastic tumor cells enclosed in a collagenous matrix, medium sized angulated vessels and enclosure of atrophic muscle fibers.

Core needle biopsies showed findings of a moderately cellular collagen-producing fascicular spindle cell proliferation of fibroblastic/myofibroblastic cells without cytologic atypia (Fig. 2). The thin-walled ectatic vessels typical of desmoid were often identified. The infiltrative growth in skeletal muscle (Fig. 2) and fascia could often be seen.

Colonoscopy and fundal examination are indicated to investigate for the presence of Gardner’s syndrome.

On ultrasonography, desmoid tumors appear as well-defined lesions with variable echogenicity. On CT scan, they may appear as homogeneous or heterogeneous and hypo-, iso-, or hyperintense compared with the attenuation of muscles [19-23]. Characteristic MRI findings include poor margination, low-signal intensity on T1-weighted images and heterogeneity on T2-weighted images, and variable contrast enhancement [19, 21, 24]. MRI is superior to CT scan in defining the pattern and the extent of involvement as well as in determining if recurrence has occurred after surgery, though both the modality CT and MRI aid in determining the extent of local invasion.

Immunostaining with vimentin, alpha smooth muscle actin, muscle actin, and desmin is helpful in distinguishing the tumors in the differential diagnosis of desmoid tumors. APC germline mutations in apparently sporadic desmoid tumor patients who have no clinical or familial signs of FAP but have a family history of colorectal carcinoma in at least one family member were evaluated by Brueckl et al [9] in 2005. They reported that patients with sporadic desmoid tumors and no clinical or laboratory signs of FAP may not need to be routinely tested for germline mutations of the APC gene. However, performing an APC mutational analysis instead of other tests (e.g., esophagogastroduodenoscopy, complete colonoscopy) may be a more time- and cost-effective plan.

### Histopathological findings

On gross examination, the tumors appear firm and smooth, without necrosis or hemorrhage. An intact capsule surrounds the periphery with initial inspection; however, the tumor characteristically extends beyond this pseudo-capsule, with fibrous septae of tumor extending radially.

On cut surface, they are gritty, glistening white and trabeculated resembling scar tissue.

**Figure 3:**
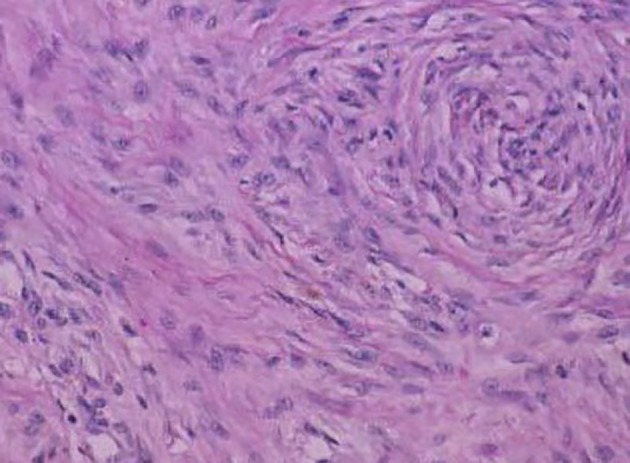
Fibrocytic cells of a desmoid tumor growing in a haphazard manner and producing collagen.

**Figure 4:**
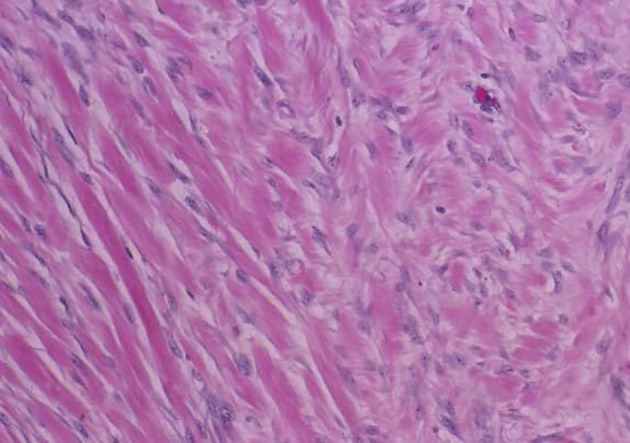
Desmoid tumor spindle cells invading skeletal muscle.

On H&E section, the tumor consists of elongated spindle cells reminiscent of fibroblasts presenting uniform appearance with eosinophilic cytoplasm embedded within a collagenous matrix. The nuclei are small, vesicular, pale staining, and sharply defined, and may contain one or two tiny nucleoli, but lack hyperchromasia. Intra-abdominal desmoids can sometimes contain relatively scanty collagen and a pronounced myxoid matrix. Spindle cells and collagen fibrils are usually arranged in ill-defined fascicles and interlacing bundles (Figs. 3, 4). Occasionally extensive glassy hyalinization, which is also a feature of keloid, may also be encountered. The tumors can vary in cellularity and may assume a more storiform architecture as well. Special stains such as reticulin and mason trichome have been traditionally used to highlight the collagen more clearly, but are currently rarely employed.

On electron microscopic examination, the spindle cells of desmoid tumors appear to be myofibroblasts. This finding is thought to represent an abnormal proliferation of myofibroblasts, which normally disappear gradually during the later stages of wound healing.

The aforementioned features are in contrast to those in a fibrosarcoma, which has greater mitotic activity, an increased nuclear-to-cytoplasm ratio, greater vascularity, less collagen production, and a paucity of immune cells [8].

Immunohistochemistry can help clarify difficult diagnoses; the tumors typically stain positive for beta-catenin, actin and vimentin and stain negative for cytokeratin and S-100.

### Management

Management of patients with desmoid tumors is difficult and many issues remain controversial, mainly regarding early detection, the role, type and timing of surgery, and the value of non-operative therapies [6]. The main difficulty in treatment is due to the fact that these tumors are histologically benign but have a high propensity for local recurrence [25]. Women have been found to be more likely to require multiple desmoid tumor resections than men, an observation which supports the hypothesis that estrogens stimulate desmoid growth [4]. Estrogen’s regulatory role is supported further by the higher incidence of desmoid tumors in women during their reproductive years, the apparent tendency of tumors to develop during pregnancy or soon after, their occasional disappearance after menopause, the proliferation of similar lesions in laboratory animals by estrogen administration and the potential benefit of anti-estrogen drugs [5, 6, 25, 26].

There are no good randomized clinical trials of treatment for desmoid tumors and most studies are based on small case series. The effects of treatment are further compounded by the variable natural history of the disease with some tumors apparently regressing or remaining stable even without treatment [6].

Management of desmoid tumors involves a multidisciplinary approach with rapidly growing tumors being managed more aggressively [25]. Patients must be managed by a team of general surgeons, a plastic surgeon, a radio-oncologist, a psychological counselor, a social worker, nurses and a pathologist.

Surgery is the mainstay of treatment in the management of extra-abdominal desmoid tumors and resection of abdominal wall tumors especially can be performed safely [3, 4]. Attempts have been made in a number of reports to define the optimal operative procedure by evaluating outcome according to the type of resection - designated variously as simple excision, local excision, wide excision, adequate resection, inadequate resection, radical local excision, and so on - without evaluating the actual pathologic margin. It is the actual status of the microscopic surgical margin that best determines the risk of local recurrence. In multivariate analysis, margin status was the single most significant determinant of recurrence in patients treated with surgery. Other studies that addressed pathologic margin status had similar findings: recurrence rates for margin-positive versus margin-negative resections have been reported as 43% vs. 15% [27], 47% vs. 15% [28], 68% vs. 12% [29], and 42% vs. 22% [30]. Radical (free margin) excision offers the best chance for cure and of avoiding local recurrence [25]. Unfortunately, radical surgery is not always a straightforward procedure because of the tumor’s extent and invasiveness. Superficial abdominal wall desmoid tumors should be resected before they become large in order to avoid having large soft tissue defects with resultant complicated and technically more demanding reconstruction [31-33]. Abdominal wall reconstruction can be achieved by direct repair (with sutures), and by using synthetic materials (meshes) or myocutaneous flaps when the defect is large [32-35]. Surgery may also be required for the management of complications such as hemorrhage, bowel perforation, hollow visceral obstruction, peritonitis or sepsis.

Radiotherapy, chemotherapy, and endocrine therapy are used in patients with inoperable tumors, local recurrences, or incompletely excised lesions [19, 24, 36, 37]. Metastatic disease has not been reported with desmoid tumor. Only few cases of malignant transformation in desmoid tumor are reported, and all were associated with local irradiation.

Radiation therapy has been used mainly for the treatment of extra-abdominal desmoid tumors and has resulted in improvement of local control of desmoid tumors by reducing local recurrence rates [34, 35, 38, 39]. External-beam irradiation or brachytherapy has been used alone in patients with inoperable lesions, but it has been associated with high failure rates [1, 35]. Radiotherapy may also be used either before surgery or as adjuvant therapy following incomplete (non-radical) surgical resection [4, 25, 40, 41]. The role of radiofrequency ablation in the management of these tumors is still under investigation and could be considered in selected patients and only when other treatment modalities have failed [39]. Percutaneous chemical ablation with acetic acid under radiological guidance is another therapeutic option and unproven treatments with pirfenidone, interferon alpha and imatinib (800 mg/day) may be effective, but only anecdotal reports or small series have been published so far [42-46]. Gene transfer therapy is also a field of intensive research currently in the management of desmoid tumors [47].

A variety of systemic agents, including tamoxifen, non-steroidal and steroidal anti-inflammatory agents, testolactone, and cytotoxic chemotherapeutic agents, have been reported to produce partial or complete tumor responses. The dogma prevalent in medical oncology has been that low-grade tumors with no known metastatic potential do not kill patients and should not respond to chemotherapy. Desmoid tumors, especially when associated with FAP, defy this dogma on both counts. These tumors are responsible for death in up to 11% of these patients, second only to colorectal carcinoma [48]. Therefore, it is important to treat appropriate patients early and aggressively with systemic therapy to avoid life-threatening complications. Clinical situations without any impending threat to life or function are usually treated with less toxic approaches, such as hormonal therapy. Tamoxifen is the most commonly used agent, with some suggestion that higher doses (up to 120 mg/day) in combination with anti-inflammatory agents are more effective than tamoxifen alone [49]. In general, the true regression rate with tamoxifen is in the 15-20% range, with another 25-30% of patients achieving symptomatic improvement with stabilization of disease, resulting in a clinical benefit rate of up to 50% [50]. Typically, these tumors are slow to manifest actual reduction in size. Not infrequently, shrinkage lags behind discontinuation of therapy by months or even years, raising the possibility that the mechanism of action may well be deprivation of the growth signal or cytokine, which results in prolonged and continued regression long after discontinuation of the therapy. This phenomenon has been noted with radiation therapy, hormonal therapy, and systemic chemotherapy.

Intra-abdominal variants, especially the ones associated with FAP, can be potentially lethal and frequently require systemic therapy. Combination chemotherapy may be highly effective in achieving significant and durable cytoreduction, obviating the need for any surgical intervention, which tends to be counterproductive in this group of patients.

### Conclusion

The history of painless abdominal mass, the age and sex of the patient, the location of the mass within the anterior abdominal wall, and the imaging features make desmoid tumor a strong primary diagnostic consideration even if it is a rare entity. Aggressive, wide surgical resection with negative margin is the best surgical option. Complete surgical excision of desmoid tumors is the most effective method of cure, sometimes necessitating removal of most of an involved anterior abdominal wall in a giant desmoid tumor. However, the radicality of resection should be tempered so that significant compromise of function is avoided. Close follow-up may be acceptable in lieu of radiation therapy if it is estimated that salvage treatment in the event of recurrence will not compromise functional and cosmetic outcome.
